# Surgical management of skull base chordomas and chondrosarcomas: insights from a national cohort study

**DOI:** 10.1136/bmjonc-2024-000386

**Published:** 2024-07-29

**Authors:** Laurence J Glancz, Cathal John Hannan, Alexandros Vyziotis, Gillian M Potter, Rekha Siripurapu, Raj K Bhalla, Scott A Rutherford, Andrew Thomas King, Charlotte Hammerbeck-Ward, Adrian Crellin, Shermaine Pan, Rovel Colaco, Gillian A Whitfield, Omar N Pathmanaban

**Affiliations:** 1 Department of Neurosurgery, Queens Medical Centre, Manchester, UK; 2 School of Medicine, University of Nottingham, Nottingham, UK; 3 Department of Clinical Oncology, The Christie NHS Foundation Trust, Manchester, UK; 4 Department of Neurosurgery, The Walton Centre NHS Foundation Trust, Liverpool, UK; 5 The Geoffrey Jefferson Brain Research Centre, Manchester, UK; 6 Division of Neuroscience, School of Biological Sciences, Faculty of Medicine, Biology and Health, University of Manchester, Manchester, UK; 7 Department of Neurosurgery, Manchester Centre for Clinical Neuroscience, Salford Royal Hospital, Northern Care Alliance, Manchester, UK; 8 Department of Radiology, Tameside and Glossop Integrated Care NHS Foundation Trust, Ashton-under-Lyne, UK; 9 Department of Neuroradiology, The Walton Centre NHS Foundation Trust, Liverpool, UK; 10 Department of Otolaryngology, Northern Care Alliance NHS Foundation Trust, Salford, UK; 11 Division of Cardiovascular Sciences, School of Biological Sciences, Faculty of Medicine, Biology and Health, University of Manchester, Manchester, UK; 12 Department of Neurosurgery, Royal Sussex County Hospital, Brighton, UK; 13 Highly Specialised Commissioning, NHS England, Redditch, UK

**Keywords:** Sarcoma, Radiotherapy

## Abstract

**Objective:**

Skull base chordoma and chondrosarcoma are distinct sarcomas of the skull base but share significant therapeutic challenges due to their proximity to critical neurovascular structures, making surgical resection difficult. We sought to establish factors associated with outcome predictors in a national cohort of patients.

**Methods and analysis:**

Data for all patients referred with a diagnosis of skull base chordoma or chondrosarcoma from April 2017 to December 2022 were obtained. We performed analyses of data pertaining to the first cohort of patients treated in the UK with proton beam therapy (PBT) to determine factors associated with obtaining gross total resection (GTR) and adequate clearance of the brainstem and optic apparatus.

**Results:**

Of 230 patients with skull base chordoma or chondrosarcoma referred for PBT, 71% were accepted for PBT, with a wide regional variation between referring neurosurgical units (29%–93%). Of the first 75 consecutive patients treated with PBT, the only factor predictive of obtaining GTR was surgical resection at a unit with higher volumes of patients accepted for PBT (OR 1.32, 95% CI 1.11 to 1.63, p=0.004). Use of intraoperative MRI (OR 4.84, 95% CI 1.21 to 27.83, p=0.04) and resection at a higher volume unit (OR 1.29, 95% CI 1.07 to 1.64, p=0.013) were associated with increased rates of tumour clearance from the brainstem/optic apparatus.

**Conclusions:**

Treatment at a higher volume centre was a key determinant of the optimal surgical outcome in this cohort. These data support the management of skull base chordomas and chondrosarcomas in higher volume centres where multidisciplinary experience can be accumulated.

**WHAT IS ALREADY KNOWN ON THIS TOPIC**

- Skull base chordomas and chondrosarcomas are distinct and rare sarcomas of the skull base that have radiological overlap and treatment parallels, including complex surgical resection and adjuvant proton beam radiotherapy. It is well established that an increased extent of resection is associated with improved outcomes in patients with chordomas and chondrosarcomas of the skull base. No national-level study has been performed to determine which factors are associated with gross total resection and adequate clearance of organs at risk to permit delivery of proton beam therapy (PBT).

**WHAT THIS STUDY ADDS**

- This national multicentre study demonstrates, for the first time, that patients undergoing resection at centres with higher surgical volumes have a better chance of obtaining a gross total resection, or adequate surgical clearance of the brainstem and optic apparatus to permit dose-escalated PBT when a gross total resection is not possible.

**HOW THIS STUDY MIGHT AFFECT RESEARCH, PRACTICE OR POLICY**

- The results of this study suggest that patients with these extremely rare malignancies have better surgical outcomes at higher-volume centres. These data should be considered when reviewing the appropriate model of care for these patients.

## Introduction

 Primary skull base sarcomas represent a considerable therapeutic challenge. Although chordomas and chondrosarcomas have distinct cells of origin and divergent natural histories, their surgical management is very similar: both are slow-growing neoplasms that typically arise from the ventral skull base, and in the modern era, they are increasingly resected using expanded endonasal approaches (EEA).^1^,^3^ Complete surgical removal is the primary treatment aim for both but this is often challenging due to their proximity to neurovascular structures at the skull base and this is compounded by disease rarity, leading to low surgical volumes. It is well established that the extent of resection influences progression-free and overall survival in patients with chordoma and chondrosarcoma.^1^,^8^

Early postoperative radiotherapy improves disease outcomes in chordoma.^4^ Similarly, to prevent local relapse in chondrosarcoma, most authors agree that surgery combined with adjuvant radiotherapy is superior to surgery alone.^9^,^13^ Both tumour types are relatively radioresistant, requiring higher doses to achieve control and exceeding the tolerance of the brainstem and optic apparatus.^3^ Particle beams such as proton beam therapy (PBT) and carbon ion therapy allow a lower exit dose, depositing the majority of their energy at their target and thus minimising damage to surrounding structures. Therefore, where gross total resection (GTR) of the tumour is not possible, maximally safe resection, including optimised clearance from critical structures, is the aim, enabling the delivery of dose-escalated particle therapy. Ideally, a 5-mm optic apparatus clearance and a 3-mm brainstem clearance are needed to avoid dose compromise or radiation injury.^14^

The first National Health Service (NHS) centre providing PBT in the UK opened in Manchester in 2018 and the first patient with skull base sarcoma was treated in February 2019. Patients with this pathology are first referred to the NHS England Proton Beam Panel, a group of national chordoma and chondrosarcoma experts who provisionally decide whether onward referral for consideration of PBT is appropriate and conforms with national professional consensus guidelines.^15^ In cases referred for small-volume macroscopic residual disease or complete resection, adjuvant PBT is generally approved, although there is no strict volume cut-off value that determines acceptance. Patients with disease in close proximity to critical structures (<3 mm to the brainstem and<5 mm to the optic apparatus), preventing dose escalation and thus nullifying the benefits of PBT, are rejected. Patients with a larger volume of residual disease felt to be clearly amenable to further resection with acceptable risks are also rejected, with further surgery recommended prior to reconsideration of PBT. Effectively, the NHS Proton Panel provides an objective measure of the adequacy of surgical resection.

Few studies have sought to establish surgical factors influencing the extent of resection, and there is no published data on factors influencing surgical clearance from dose-limiting structures.^5 7 16^ In this study of the first UK national cohort of patients with skull base sarcoma accepted for PBT, we sought to review surgical strategies and establish factors predictive of GTR. In addition, we established which factors were associated with adequate tumour clearance from dose-limiting structures, permitting the delivery of PBT.

## Materials and methods

Two separate cohorts of patients were analysed. For the first cohort, data were obtained from the NHS England Proton Beam Panel, pertaining to all referrals for patients with skull base chordoma or chondrosarcoma received between April 2017 and December 2022, broken down by referring centre (18 referring centres in total). The second cohort included all patients accepted for PBT for skull base chordoma or chondrosarcoma at the Christie Proton Beam Centre from February 2019 to February 2022 (10 referring centres in total). A retrospective case review was undertaken, including original operative records and detailed oncological, surgical and radiological notes from multidisciplinary team meetings. Surgical resection was performed using either EEA, whereby the ventral skull base is accessed via the nasal cavity using an endoscope and extended instruments, or open surgical approaches to the skull base involving a craniotomy and the use of an operating microscope. Readers are directed to the cited manuscripts for a more detailed description of these surgical approaches.^17 18^ Details of the surgical approach used, broken down by anatomical location, are provided in the online supplemental material.

**Figure 1:**
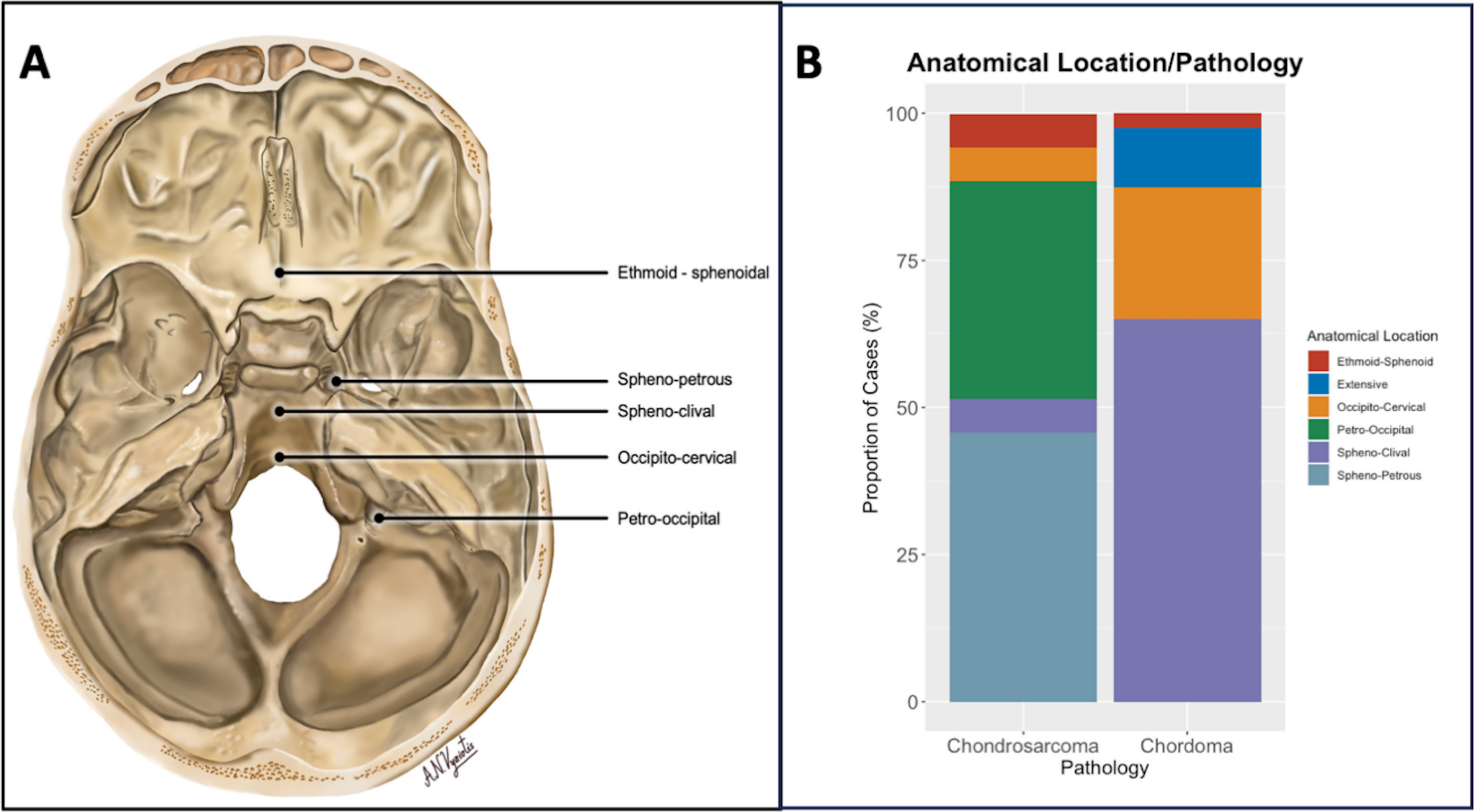
(**A**) Illustration demonstrating the anatomical classification of tumour location used for this study, as previously described by Wang *et al*.^5^ (1) Sphenoclival type—tumour in the upper two-thirds of the clivus, main body in the middle line, anterior to the sphenoid sinus and posterior to the prepontine cistern. (2) Occipitocervical type—tumour in the lower one-third of the clivus and the foramen magnum, main body in the middle line, anterior to the pharynx and posterior to the premedullary cistern. (3) Sphenopetrosal type—tumour in the parasellar or petrous apex, main body in the middle fossa, medial to the cavernous sinus and intracavernous segments of the carotid artery displaced medially or laterally. (4) Petrooccipital type—tumour in the posterior fossa laterally, from the lateral side of the Meckel cavity to the cerebellopontine angle and to the jugular foramen. (5) Ethmoid-Sphenoid type—tumour in the anterior fossa and posterior to the pituitary fossa and (6) extensive type (not shown)—tumour with a huge volume involving at least two of the five parts mentioned above. (**B**) Stacked column graph illustrating the relationship between pathology and anatomical location, as classified by classifications of tumour location. As expected, the majority (35/40, 88%) of chordomas were located in the sphenoclival or occipitocervical regions. A significant proportion of chondrosarcomas (29/35, 82%) were located in the spheno-petrous region or spheno-occipital region.

All pathological diagnoses were made in the local referring centres, with second opinions from specialist sarcoma pathologists obtained when necessary. All cases referred underwent testing for the expression of the brachyury transcription factor.^19^ Lesion location was defined by a previously reported classification system^5^ (figure 1). Sphenoclival (SC), occipitocervical (OC) and ethmoid-sphenoid tumours were defined as having a midline location, whereas spheno-petrous (SP) and petrous-occipital (PO) tumours were defined as lateral. All radiological studies were reviewed by two consultant neuroradiologists with subspecialist skull base experience (RS and GP).

All statistical analyses were performed in R statistical software (R Core Team 2023, Austria). Intergroup differences in continuous variables were assessed for statistical significance using the Student’s t-test, and differences in the distribution of categorical variables were assessed using the χ^2^ test. Univariate and multivariate regression models were used where appropriate. Alpha was set at 0.05 and only variables found to be significant on univariate analysis were included in multivariate models as potential predictor variables. This work was performed according to the Strengthening the Reporting of Observational Studies in Epidemiology guidelines.^20^ Relevant approvals were obtained and the requirement for individual patient consent was waived in view of the multicentre, anonymised nature of the data analysed. There was no public or patient involvement in this study.

## Results

### National referrals to the Proton Beam Panel

**Figure 2:**
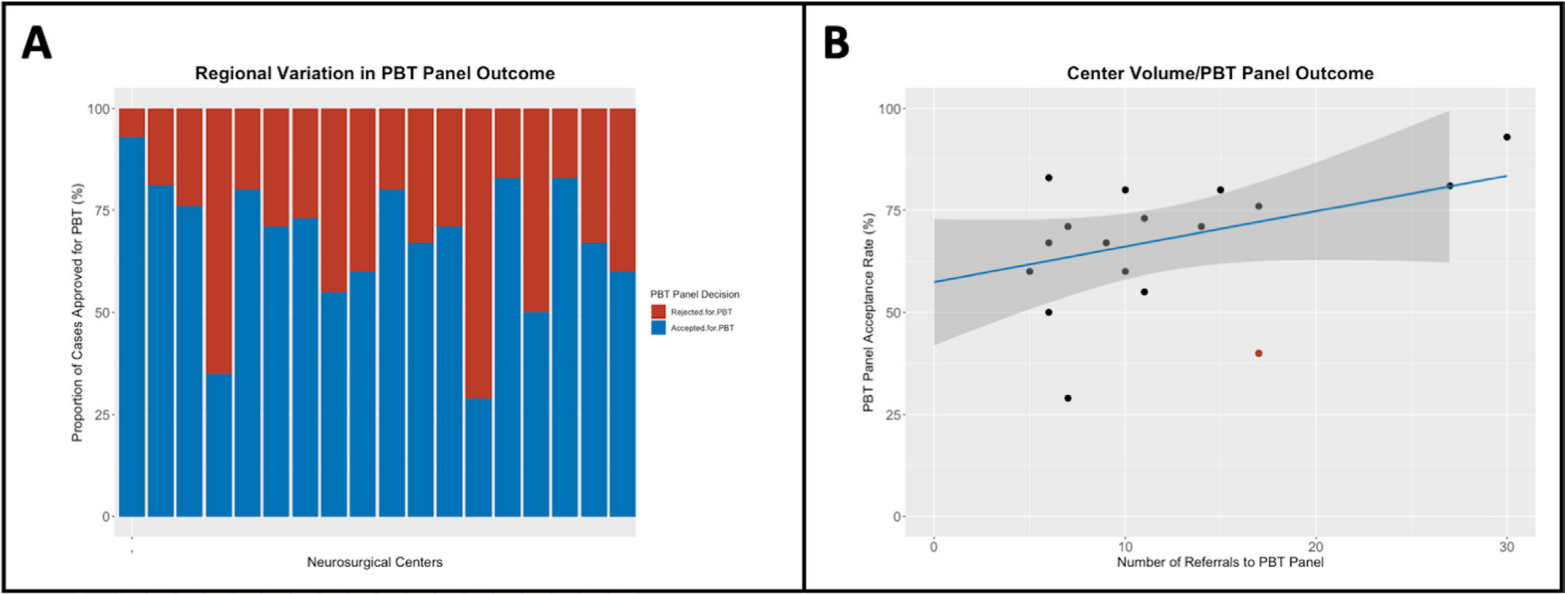
(**A**) Stacked bar graph demonstrating the wide regional variation in outcomes following surgery for skull base sarcoma; the acceptance rate ranged from 29% to 93% (**B**) Scatterplot with regression line demonstrating the colinearity between the number of referrals from each centre and the proportion of cases accepted for PBT. An outlying high-volume, low-acceptance rate centre is highlighted in red. 95% CI of the regression coefficient is indicated by grey shading.

Between April 2017 and December 2022, 230 patients with skull base chordoma or chondrosarcoma were referred from across the UK to the NHS England Proton Beam Panel. Each of the 18 referring centres was a tertiary neurosurgical centre in the UK, referring patients for consideration of PBT following resection of a skull base chordoma or chondrosarcoma. Overall, 163/230 (71%) of patients were accepted for PBT, although wide regional variations were observed (29%–93%). The relationship between the overall number of referrals to the PBT panel from each centre and the proportion of cases from that centre was not a significant predictor of acceptance (R^2^=0.13, p=0.14) (figure 2). However, this result was primarily driven by a single outlying high-volume centre with low acceptance rates, and the removal of this centre from the analysis led to the relationship between increasing volume and chance of acceptance for PBT becoming statistically significant (R^2^=0.26, p=0.03). A detailed breakdown of the referral outcomes from each centre is provided in the online supplemental material.

### Cohort accepted for PBT

#### Demographics

75 patients were accepted for PBT between February 2019 and February 2022. Their demographic details are described in table 1. 40/75 (53%) had chordoma, and 35/75 (47%) had chondrosarcoma. Of all patients accepted for proton beam, 21/75 (28%) underwent more than one resective operation; 13/75 (17%) underwent two operations, with the remainder (8/75, 11%) having between three and five resective operations. Patients with chordoma were more likely than those with chondrosarcoma to have an endonasal approach to their tumour (35/40, 88% vs 18/35, 51%, p=0.002) and were also more likely to have a GTR (23/40, 58% vs 6/35,17%, p≤0.001). The median number of cases accepted for PBT in each centre was three (range 1–14).

**Table 1:** Patient demographics

Characteristics	Chordoma (n=40)	Chondrosarcoma (n=35)	Total (n=75)	Significance
Female (%)	23 (58)	20 (57)	43 (57)	0.999
Mean age (SD)	45 (18)	51 (16)	48 (18)	0.455
Mean tumour volume (SD)	18.69 cm^3^ (20.86)	20.10 cm^3^ (18.72)	19.34 cm^3^ (19.79)	0.381
<5 mm Preoperative distance to optic apparatus (%)	14 (35)	12 (34)	26 (34)	0.948
<3 mm preoperative distance to brainstem (%)	30 (75)	27 (77)	57 (76)	0.828
Preresection biopsy (%)	4 (10)	5 (14)	9 (12)	0.568
Total number of operations (%)				
1	31 (78)	23 (65)	54 (72)	0.267
2	6 (15)	7 (20)	13 (17)	
3	2 (5)	1 (3)	3 (4)	
4	1 (3)	3 (9)	4 (5)	
5	0 (0)	1 (3)	1 (1)	
Surgical approach in primary operation				
Infratentorial	1 (3)	10 (29)	11 (15)	0.002
Supratentorial	3 (8)	7 (20)	10 (13)	
Transnasal	35 (88)	18 (51)	53 (70)	
Transoral	1 (3)	0 (0)	1 (1)	
Image guidance in primary operation (%)	29 (73)	25 (71)	54 (72)	0.931
Intraoperative MRI in primary operation (%)	8 (20)	3 (9)	11 (15)	0.162
Extent of resection following initial surgery (%)				
Gross total	23 (58)	6 (17)	29 (38)	<0.001
Subtotal	17 (42)	29 (83)	46 (61)	

Descriptive statistics of basic demographic details, underlying histology, tumour location and details of surgical approaches for all patients in the cohort are provided. Bold typeface indicates a statistically significant comparison.

The anatomical locations of the tumour, broken down by histological diagnosis, are presented in figure 1. The majority of chordomas were midline; 26/40 (65%) were SC and 9/40 (22.5%) occipitocervical (OC). Conversely, the majority of chondrosarcomas were laterally placed; 16/35 (46%) were SP and 13/35 (37%) were PO. The majority (53/75, 71%) of primary approaches taken were endoscopic endonasal, with the remainder (22/75, 29%) of primary approaches being lateral ‘open’ approaches. All 28 midline SC tumours and three in the ethmoid-sphenoid region were operated on via an endoscopic endonasal route, while those in the OC, PO and SP regions were resected via a roughly equal split of endoscopic endonasal approaches and more traditional open skull base approaches (online supplemental material).

#### Surgical outcomes: extent of resection

To assess factors associated with obtaining a GTR and achieving adequate surgical clearance of the brainstem and optic apparatus, data obtained from the cohort accepted for PBT (n=75) were analysed. Following the review of postoperative imaging, a GTR was achieved in 29/75 (38.6%) of patients who received PBT. Factors associated with achieving GTR were assessed using univariate logistic regression models and chordoma diagnosis (vs chondrosarcoma) (OR 8.33, 95% CI 2.58 to 33.01), use of EEA (OR 7.33, 95% CI 1.81 to 49.08), midline tumour location (OR 12.97, 95% CI 3.20 to 88.53) and an increasing number of cases accepted for PBT (OR 1.23, 95% CI 1.07 to 1.43) were all significantly associated with obtaining a GTR. Age, tumour volume, use of intraoperative image guidance and intraoperative MRI (iMRI) did not influence the extent of resection (table 2).

**Table 2:** Factors associated with gross total resection/clearance of organs at risk

Factor	Univariate analysis			Multivariate analysis		
	OR	95% CI	Significance	OR	95% CI	Significance
Gross total resection						
Age	0.73	0.35 to 1.30	0.557			
Chordoma	**8.33**	**2.58 to 33.01**	**<0.001**	5.60	0.66 to 70.86	0.137
Expanded endonasal approach	**7.33**	**1.81 to 49.08**	**0.013**	1.89	0.23 to 20.41	0.558
Image guidance	1.8	0.56 to 6.42	0.334			
Intraoperative MRI	2.4	0.63 to 9.44	0.194			
Midline location	**12.97**	**3.20 to 88.53**	**0.002**	2.49	0.17 to 36.95	0.491
Number of cases accepted for PBT	**1.23**	**1.07 to 1.43**	**0.004**	**1.32**	**1.11 to 1.63**	**0.004**
Tumour volume	0.98	0.94 to 0.99	0.245			
Adeqaute clearance of organs at risk						
Age	1.02	0.38 to 2.77	0.968			
Chordoma	**5.47**	**1.26 to 38.34**	**0.041**	0.38	0.04 to 2.73	0.369
Expanded endonasal approach	**4.04**	**1.29 to 14.44**	**0.021**	2.13	0.28 to 23.34	0.481
Image guidance	1.50	0.50 to 4.63	0.471			
Intraoperative MRI	**2.76**	**1.02 to 7.86**	**0.049**	**4.84**	**1.21 to 27.83**	**0.04**
Midline location	2.67	0.93 to 7.98	0.071			
Number of cases accepted for PBT	**1.21**	**1.05 to 1.43**	**0.009**	**1.29**	**1.07 to 1.64**	**0.013**
Tumour volume	**0.97**	**0.93 to 0.99**	**0.034**	0.99	0.95 to 1.02	0.678

Results of statistical analyses assessing factors potentially associated with gross total resection and clearance of brainstem and optical apparatus in the cohort of patients accepted for PBT (n=75). Bold typeface indicates a statistically significant comparison. Chordoma diagnosis, midline tumour location, midline anterior surgical approaches and an increasing number of patients accepted for PBT were significantly associated with increased rates of gross total resection on univariate analysis. Following multivariate analysis using a logistic regression model, an increasing number of patients accepted for PBT was the only significant predictor of obtaining a gross total resection. Decreasing tumour volume, chordoma diagnosis, endoscopic endonasal surgical approaches, an increasing number of cases accepted for PBT and the use of intraoperative MRI were significantly associated with increased rates of clearance of organs at risk. Following multivariate analysis, the use of intraoperative MRI and the increasing number of cases accepted for PBT were the only significant predictors of obtaining adequate clearance of the brainstem and optic apparatus.

PBT, proton beam therapy.

**Figure 3:**
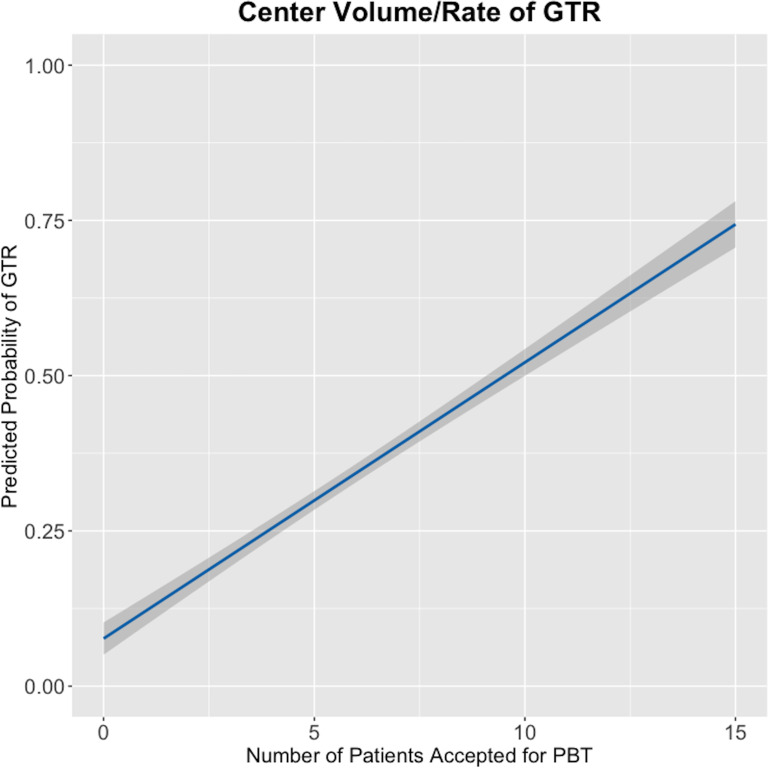
Graphical representation of a linear regression model demonstrating the influence of the number of patients accepted for PBT on the predicted probability of obtaining a GTR during resection of a chordoma/chondrosarcoma. The probability of achieving GTR increased linearly as the number of patients per unit accepted for PBT increased. 95% CI of the regression coefficient is indicated by grey shading. GTR, gross total resection; PBT, proton beam therapy.

On multivariate analysis, the only factor found to significantly impact the rate of GTR was the volume of cases accepted for PBT; for each additional case per unit accepted for PBT, the probability of obtaining a GTR increased by 32% (OR 1.32, 95% CI 1.11 to 1.63) (figure 3)

#### Surgical outcomes: adequate clearance of dose-limiting structures

Variables associated with a significantly increased probability of clearance of vital structures in the primary operation included chordoma diagnosis (OR 5.47, 95% CI 1.26 to 38.34), use of EEA (4.04, 95% CI 1.29 to 14.44), use of iMRI (OR 2.76, 95% CI 1.02 to 7.86), an increasing number of patients accepted for PBT (OR 1.21, 95% CI 1.05 to 1.43) and decreasing tumour volume (OR 0.97, 95% CI 0.93 to 0.99). Multivariate logistic regression was performed to ascertain the effects of the aforementioned significant factors on univariate analysis on the likelihood of obtaining clearance of vital structures (table 2). On multivariate analysis, surgical resection with adjunctive iMRI (OR 4.84, 95% CI 1.21 to 27.83) and an increasing number of patients accepted for PBT (OR 1.29, 95% CI 1.07 to 1.64) were significantly associated with an increased chance of achieving adequate clearance of the brainstem and optic apparatus.

## Discussion

### Summary of results

In this first national-level study of patients with skull base chordoma and chondrosarcoma, we have reviewed regional rates of achieving criteria for PBT acceptance and determined which factors are associated with optimal surgical outcomes in the initial cohort of patients treated in the UK PBT Programme. We identified substantial regional variations in the proportion of patients who were deemed suitable for PBT; the rate of acceptance following the national PBT panel review ranged from 29% to 93%, depending on the referring centre. Moreover, although we did not identify a significant relationship between overall referral numbers (and therefore the volume of resected chordomas and chondrosarcomas) and the proportion of cases from that unit accepted for PBT, we did identify a clear trend towards cases from higher-volume centres being accepted for this treatment. This was with the exception of a single high-volume centre with low rates of acceptance, which skewed the results (figure 2). This underlines the fact that high volume alone does not guarantee optimal surgical outcomes.

When we assessed a more selected group of 75 patients accepted for PBT and treated in the first UK PBT centre, we identified a statistically significant volume–outcome relationship. In this cohort, we have demonstrated a clear relationship between surgical volume and outcome: for each additional case accepted for PBT per neurosurgical unit, the chance of a patient treated there obtaining a GTR increased by 32% (table 2 and figure 3). We demonstrated the same relationship when we assessed factors associated with adequate clearance of the brainstem and optic apparatus to facilitate the delivery of PBT: surgical resection at a higher volume unit and use of iMRI were both significantly associated with achieving sufficient clearance of these organs at risk.

### Results in the context of the literature

Our results demonstrated a large and statistically significant effect of volume on surgical outcome: the chances of obtaining a GTR were significantly greater in neurosurgical units with higher surgical volumes (table 2, figure 3). These results are not surprising; in other technically challenging areas of neurosurgery, such as giant pituitary adenomas, vestibular schwannomas and aneurysmal subarachnoid haemorrhage, a clear relationship between surgical case volume and outcome has been demonstrated previously.^21^,^23^ Our study is the first to demonstrate such a relationship for skull base sarcomas, and these results are concordant with those of a US national registry study demonstrating the same finding for spinal chordomas.^24^ Moreover, a recent study of all UK patients with retroperitoneal sarcoma reported very similar findings to those of the present study: those patients managed in higher-volume centres had significantly better surgical and oncological outcomes.^25^

We found the use of endoscopic endonasal approaches were associated with a higher GTR rate on univariate analysis, along with midline tumour location and chordoma diagnosis. These results are not unexpected, given they are interrelated. The endoscopic endonasal route has become more popular over recent years and allows for direct access to midline lesions with improved visualisation and reduced morbidity.^26^ A multicentre study of 182 cases of clival chordoma found that the use of the endoscopic endonasal approach was associated with fewer complications, a higher rate of GTR and prolonged overall survival.^27^ These results were supported by a more recent systematic review focusing on the outcomes following surgical resection of chordomas and chondrosarcomas; the endoscopic endonasal approach was associated with less morbidity and higher rates of resection.^28^ However, tumours centred within the lateral skull base pose a greater challenge for GTR via this approach, due in large part to the increased difficulty in obtaining access lateral to the carotid artery via an endonasal approach.^29 30^ Although endoscopic approaches permitting access to this region are routinely used in a few UK centres, they have yet to be more widely adopted within UK neurosurgical practice.^31 32^ This is reflected in our results; tumours situated more laterally within the SP or PO regions were more likely to be resected via open transcranial approaches (online supplemental material).

Some of our findings have been reported in other series to date. Bai *et al* reported on 284 skull base chordomas and found that revision surgery, larger tumour volume and tumour location in the lower clivus were associated with a lower GTR rate.^1^ They initially reported no difference between different approaches taken when comparing endoscopic endonasal versus microscopic open lateral approaches, although, in a later series, much better outcomes were reported in their more recent endonasal cohort.^13^ Koutourousiou *et al* reported on their chordoma group operated over two time periods, with very similar findings: tumour volume >20 cm^3^, tumour location in the lower clivus with lateral extension and previously treated disease were all limitations for GTR; however, the most significant factor was the learning curve, with much higher rates of GTR in their later operated group.^29^ This latter finding is mirrored to some extent in our results, whereby higher-volume centres with more surgical experience were found to have higher rates of GTR, although we have demonstrated this on a national, multicentre basis for the first time in the present study. Freeman *et al* also showed that early access to a multidisciplinary centre resulted in better outcomes for patients with clival chordoma.^33^ Initial management in a multidisciplinary centre resulted in a significant improvement in PFS versus initial surgery with or without radiotherapy outside of this setting. Initial surgical resection outside of a multidisciplinary setting increased the risk of recurrence or progression in both univariate and multivariate analyses.^33^ Similarly, a recently published analysis of the introduction of national reference centres for sarcoma in France demonstrated a significant increase in the overall survival of patients who were managed inside a specialist centre with access to the full range of multidisciplinary expertise. The authors attributed this increase in survival to the better surgical outcomes obtained in the reference centres: a GTR was obtained in 60.6% of patients undergoing surgical resection in a reference centre, compared with only 34.6% of patients outside these centres, and those undergoing initial surgery in a reference centre were much less likely to require further surgical resection.^34^

To our knowledge, this is the first study assessing factors associated with adequate surgical clearance of dose-limiting structures in patients with skull base sarcoma considered appropriate for PBT. Interestingly, the use of iMRI was found to be a significant predictor of obtaining clearance from dose-limiting structures on multivariate analysis, alongside greater surgical experience. This finding is concordant with others describing the outcomes with iMRI as an adjunct to pituitary surgery; several case series and a recent meta-analysis have demonstrated increased rates of GTR with this technique.^35^,^38^ iMRI is also used to ensure that the optic apparatus is sufficiently decompressed in cases where GTR is not deemed to be possible.^39^ However, in this study, the vast majority of iMRI cases were performed in a single high-volume centre, which may limit the generalisability of this result. Nevertheless, in our experience, the use of iMRI is extremely useful in selected cases, ensuring adequate clearance from structures at risk of significant radiation toxicity, even where a GTR is not feasible, for example, in multiply operated fields with scarring and a high risk of morbidity with GTR. Immediate review of intraoperative images permits further resection as required while the patient remains under anaesthesia.

### Limitations

Although the UK has a socialised healthcare system with an assumed high case ascertainment, our patient cohorts are to some degree selected; those patients with the worst surgical outcomes may not have been referred for PBT. Therefore, it may be the case that the proportion of patients eligible for PBT in certain areas may be even lower than that demonstrated in figure 2. Equally, however, it is conceivable that some patients operated with GTR were not referred for PBT, especially if the tumour was a low-grade chondrosarcoma. Moreover, we were only able to perform a detailed analysis of the factors associated with GTR/clearance of critical dose-limiting structures in those patients accepted for PBT where we had complete case records and imaging available, irrespective of the institution where surgery or imaging was performed.

We also deliberately have not assessed longer-term oncological outcomes, as this would be inappropriate given the divergent clinical phenotypes of chordoma and chondrosarcoma. The primary focus of this paper was to assess factors associated with surgical outcomes, principally the extent of resection. The majority of iMRI cases were done in a single high-volume centre, and this collinearity between high surgical volume and use of iMRI may have skewed the results of the regression model assessing factors associated with adequate clearance of dose-limiting structures.

In this first national study of patients with skull base chordoma or chondrosarcoma, we have demonstrated a significant relationship between centre volume and surgical outcome. The use of iMRI was associated with an increased probability of achieving clearance of critical dose-limiting structures, enabling appropriate dose-escalated PBT. These data support the management of patients with skull base chordoma and chondrosarcoma in higher-volume centres with appropriate surgical and radiological experience, helping optimise overall patient outcomes.

## Supplementary material

10.1136/bmjonc-2024-000386 online supplemental file 1

## Data Availability

Data are available upon reasonable request.
