# Supplementary material for: Surgical management of skull base chordomas and chondrosarcomas: insights from a national cohort study
Source: BMJ Oncol. 2024 Jul 29;3(1):e000386. doi: 10.1136/bmjonc-2024-000386 (PMC11347697; doi:10.1136/bmjonc-2024-000386)
Supplement: online supplemental file 1 [file bmjonc-2024-000386supp001.pdf]

Referring Neurosurgical Centre	**No**	**Yes**	**Total**	**% Acceptance**
1	2	28	30	93
2	5	22	27	81
3	4	13	17	76
4	11	6	17	35
5	3	12	15	80
6	4	10	14	71
7	3	8	11	73
8	5	6	11	55
9	4	6	10	60
10	2	8	10	80
11	3	6	9	67
12	2	5	7	71
13	5	2	7	29
14	1	5	6	83
15	3	3	6	50
16	1	5	6	83
17	2	4	6	67
18	2	3	5	60

Supplementary Table 1. Table detailing the numbers of cases referred to the NHS Proton Panel between April 2017 and December 2022, alongside the rates of acceptance/rejection for PBT

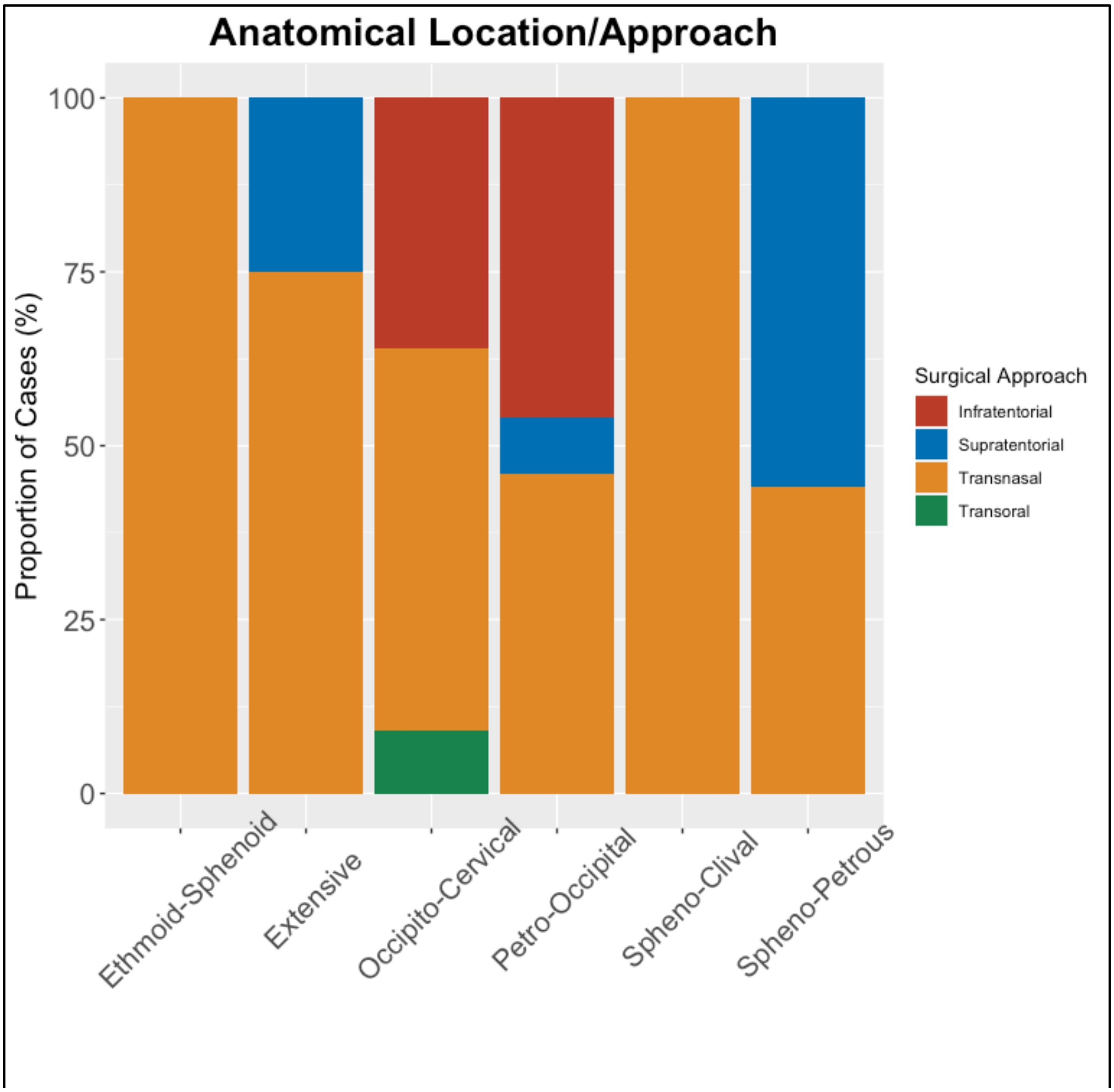

Supplemental Figure 1. Stacked column graph illustrating the relationship between anatomical location and surgical approach. All tumors in the sphenoid-clival and sphenoid-ethmoid location, alongside the majority of tumors in the occipito-cervical region (6/11, 55%) were operated on via a transnasal approach. Conversely, the majority of petro-occipital (7/13, 54%) and spheno-petrous (9/16, 56%) were approached using more traditional open skull base approaches
